# Handwritten tax receipt recognition method based on improved DBNet and CRNN

**DOI:** 10.1371/journal.pone.0352963

**Published:** 2026-07-07

**Authors:** Lan Wei, Jingqi Sun

**Affiliations:** 1 School of Finance and Economics, Sanya University, Sanya, China; 2 College of Educational Arts and Sciences, National University of the Philippines, Manila, Philippines; University of Baghdad, IRAQ

## Abstract

To recognize handwritten tax invoice text and manage tax invoices, a handwritten tax invoice recognition method based on improved differentiable binary network and convolutional recurrent neural network (CRNN) is proposed. This method first achieves bill text detection and localization through a differentiable binary network that integrates feature pyramid enhancement modules (FPEM). An improved CRNN is used to achieve bill text content recognition. In terms of text detection, the proposed module has an average detection precision of 89.1%, a recall rate of 86.4%, and an average frame rate of 63.5 frames per second. Compared with the Text Detection model based on Segformer and Enhanced Feature Pyramid and the Improved Fourier Contour Embedding Network, the precision has increased by 4.9% and 3.5%, respectively, and the frame rate has increased by 20.5 and 11.7 frames per second. In terms of text recognition, the improved CRNN has an average recognition precision of 95.6%, a recall rate of 94.2%, an average frame rate of 56.5 frames per second, and an F1-Score of 0.94. Compared with the Dual-Stream Network Fusing Spatial and Frequency Domain Feature and the Multi-modal Network based on Visual Attention and Semantic Perception, the precision has been improved by 4.9% and 3.0%, respectively, and the F1-Score has been improved by 0.07 and 0.04. This method performs excellently in processing complex handwritten tax receipts, with outstanding recognition precision and robustness, significantly reducing tax workload, and improving tax declaration and management efficiency.

## 1. Introduction

Driven by information technology, the digital transformation of tax management has become an important means to improve tax declaration efficiency and management level. Handwritten tax receipts are important vouchers in tax management, and their fast and accurate digital input is a key link in achieving efficient tax management [1,2]. However, traditional manual input methods are not only inefficient, but also prone to errors, greatly limiting the modernization process of tax management. Therefore, developing an automated handwritten tax receipt recognition method has important practical significance. In recent years, Optical Character Recognition (OCR) has made significant progress in document processing and information extraction. OCR technology automatically recognizes and parses text content in images through feature extraction, text detection, and character recognition, and converts it into editable and searchable data formats. OCR technology can quickly recognize and extract textual information from documents, significantly reducing the workload of manual input. However, handwritten tax receipts face many challenges. The differences in handwritten fonts are significant, there may be serious linking and interference between characters, and the layout of the receipts is complex and diverse, all of which increase the recognition difficulty [3,4]. Therefore, developing an efficient and accurate handwritten tax receipt recognition method is currently a hot and difficult topic.

For text detection, Mansouri et al. proposed an automatic detection method that combined edge information and maximum stable extremum regions for Arabic text detection and localization in video frames. This method extracted text region candidates and grouped and filtered these regions based on geometric attributes such as area and direction. The method performed satisfactorily in a large number of Arab TV news tests [5]. Qin and Chen integrated bottom-up and top-down methods for text detection in arbitrarily shaped scenes. This method used segmentation as a bottom-up detector to regress text regions, and an anchor free method as a top-down detector to distinguish each text. The method achieved better performance on multiple common benchmarks, including regular shapes and arbitrary shapes [6]. Liang et al. decoupled feature pyramid network and regression links deal with the text detection in arbitrarily shaped scenes. This method separated the width and height of the feature map by decoupling the feature pyramid network. The regression linking was used to link pixels to text instances to expand the detection ability of the rotated rectangular text detector for curved text. This method could effectively detect scene text of any shape [7]. Khan and Mollah proposed a two-stage text detection method based on edge point neighborhood and deep network for scene text detection in Hindi environment. This method generated fine scale edge maps from the original image, then used adaptive clustering to form clusters based on edge point density, extracted foreground objects as text proposals, and performed text detection through deep Convolutional Neural Network (CNN). The proposed method outperformed the existing methods on the benchmark dataset [8].

For handwritten text recognition, He et al. built a text inference method based on visual semantics to deal with the shortcomings of existing scene text recognition methods in handling arbitrary shaped text. This method constructed sub-graphs and merged them into a complete graph, using graph convolutional networks for text inference. The results showed that the model constructed in parallel with graph convolutional networks and language models exhibited generalization ability on multi-lingual datasets [9]. Souibgui et al. built a text degradation invariant auto-encoder method for text recognition and document image enhancement. This method adopted a Transformer-based and did not use labeled data during pre-training. This method surpassed existing methods in handwriting and scene text recognition, as well as document image enhancement [10]. Barrere et al. built a lightweight Transformer to address the data scarcity and difficulty in generating synthetic data in historical handwritten text recognition. This method adopted realistic synthetic data to reproduce historical handwriting, while designing a specialized training and prediction strategy to address the limited training data. The text recognition could surpass existing methods [11].

In a word, although some progress has been achieved in current handwritten text recognition technology, it still faces some limitations. Firstly, the diversity and complexity of handwritten text pose significant challenges to handwritten text recognition. The writing styles vary greatly, and the shape, size, tilt angle, and stroke coherence of fonts may vary from person to person. This makes it extremely difficult to build a universal model that can accurately recognize various handwriting styles. In addition, handwritten text often has connected strokes, blurring the boundaries between characters, and further increasing the recognition difficulty. This linking phenomenon not only varies from person to person, but may also differ due to factors such as writing speed and writing habits, making it difficult for the model to accurately segment and recognize individual characters. In summary, existing methods still have obvious technical shortcomings: most text detection models lack fusion of handwritten text features that are bent or tilted, making it difficult to balance positioning accuracy and detection speed. The handwriting recognition model has poor adaptability to character adhesion and font variations, and the feature extraction ability of a single network is limited, which cannot fully capture the complex text features of handwritten tax receipts. Existing OCR solutions are mostly designed for general scenarios and do not take into account the fixed layout and professional character characteristics of tax receipts, which greatly reduces the actual recognition effect. Based on the aforementioned research gaps, this study combines the detection advantages of Differential Binarization Network (DBNet) with the sequence recognition advantages of CRNN to make targeted improvements, aiming to create a high-precision and highly robust recognition method suitable for handwritten tax bill scenarios. DBNet is a deep learning model for text detection, which is particularly suitable for scene text detection tasks. It achieves efficient and accurate text detection by combining binary operations and differentiable design. CRNN combines CNN and Recurrent Neural Network (RNN). It can extract image features through CNN and then use RNN to model sequence information, thereby achieving efficient and accurate sequence recognition. Therefore, to improve the OCR recognition accuracy and accurately recognize handwritten tax receipts, a handwritten tax receipt recognition method based on DBNet and CRNN is built. The innovation and contribution of the research are as follows: (1) Improving the DBNet detection model: Fusing the FPEM with deformable convolution to solve the insufficient feature fusion and detail loss in the original DBNet, achieving a better balance between detection speed and accuracy. (2) Optimizing the CRNN recognition model: Replacing the original CNN convolutional layer with DenseNet, preserving width information through a 1*2 average pool, and capturing bidirectional sequence dependencies using a dual-BiLSTM to improve the recognition robustness of cursive and diagonal handwritten fonts. (3) Building a dedicated tax invoice recognition framework: Solving the large differences in handwritten fonts and complex layouts of tax invoices, and achieving integrated detection and recognition. Compared with general OCR methods, the processing efficiency and accuracy in tax invoice scenarios are greatly improved, providing an efficient technical solution for digital tax management.

The first section is an introduction, which elaborates on the research background and challenges of handwritten tax bill recognition, sorts out the relevant research status, and proposes the recognition method and innovation points based on improved DBNet and CRNN. Section 2 is about methods and materials, which respectively introduce the structure and principles of the improved DBNet detection model and the optimized CRNN recognition model that integrate FPEM. Section 3 presents the experimental results to validate the performance of the recognition model, conducts comparative and ablation experiments, and analyzes the data. Section 4 discusses and analyzes the reasons for the performance advantages of the model, and compares existing methods to demonstrate the value. Section 5 is the conclusion, summarizing the research results, pointing out the limitations of the model, and looking forward to future research directions.

## 2. Methods and materials

To optimize the efficiency of tax declaration and management, digitally manage tax receipts, a handwritten tax receipt recognition method based on DBNet and CRNN is built. This method first uses DBNet-FPEM to detect the text of handwritten tax receipts, and then uses an improved CRNN to recognize the content of handwritten tax receipts.

### 2.1. Handwritten tax receipt detection method based on DBNet and feature pyramid enhancement

**Fig 1:**
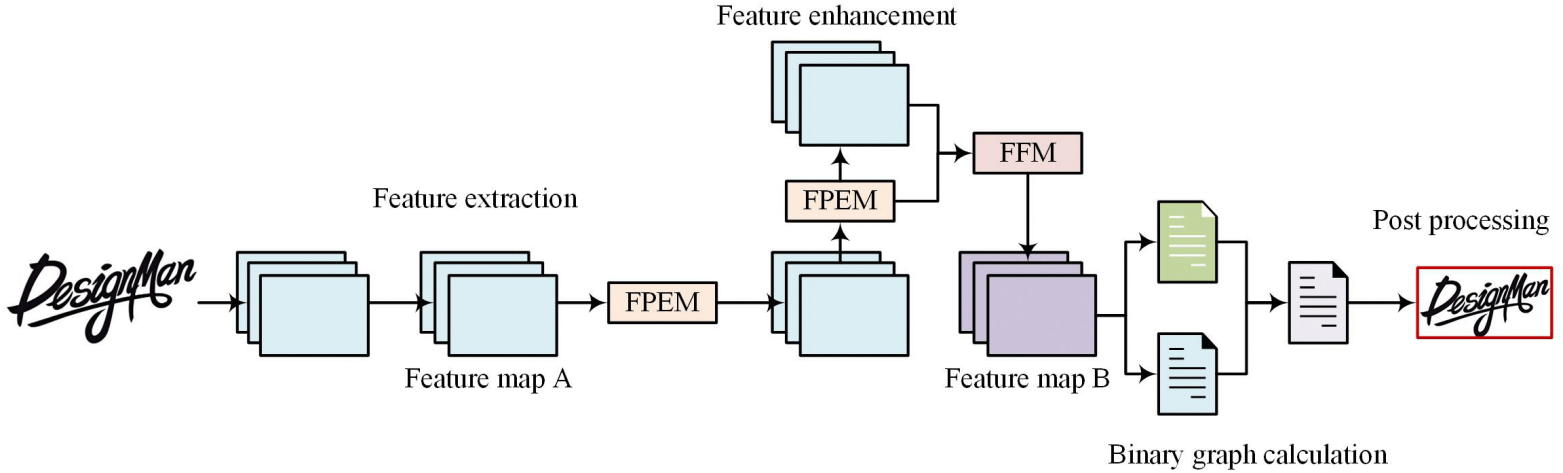
Handwritten tax receipt detection model based on DBNet-FPEM.

With the application and promotion of OCR technology, handwritten tax receipts can be quickly recognized and extracted, without the need for manual input one by one, greatly reducing the workload. Meanwhile, OCR technology can support batch recognition and processing of multiple receipts. Even if they are mixed together, they can be automatically segmented and recognized, further improving the receipt processing efficiency. However, due to the significant font differences and serious interference between characters in handwritten tax receipts, their detection is difficult [12,13]. Therefore, to accurately detect handwritten tax receipts, a DBNet-based handwritten tax receipt detection method is proposed to accurately locate handwritten text. DBNet is a neural network architecture that combines binary operations and differentiable design, which has high computational efficiency and detection accuracy [14,15]. However, due to the limitation of feature fusion, much detailed information is often lost, occurring errors in handwritten text localization. Therefore, to improve the text positioning accuracy of handwritten tax receipts, FPEM and feature fusion modules are introduced to improve DBNet. The handwritten tax receipt detection model based on DBNet-FPEM is presented in Fig 1.

**Fig 2:**
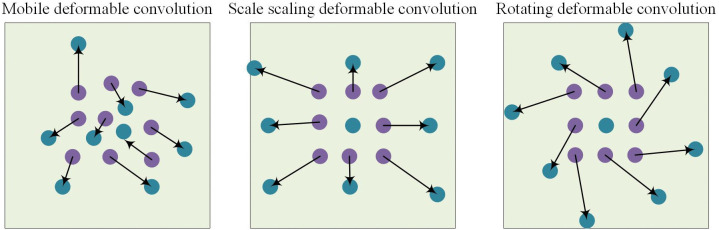
Deformable convolution.

From Fig 1, in the feature extraction stage, the model first uses a Residual Network (ResNet) to extract the features of handwritten tax receipts. In the feature enhancement stage, the model enhances the feature map through FPEM to optimize the expression ability. At the binary graph calculation node, the model divides the pixels into text and non-text regions to clearly identify the text. In the post-processing stage, the model performs binary operations on the obtained probability map and performs segmentation processing on the binary map to accurately locate the text region. In the DBNet-FPEM model, ResNet is used for feature extraction. However, due to the influence of the receptive field of ordinary convolutional blocks, it is difficult to capture features of different sizes. Therefore, the study introduces deformable convolution to replace ordinary convolution. Fig 2 displays the deformable convolution.

In Fig 2, the deformable convolutions include mobile deformable convolution, scale scaling deformable convolution, and rotating deformable convolution. The mobile deformable convolution allows the convolution kernel to undergo deformation during the convolution process to better adapt to local changes in the input data. Scale scaling deformable convolution increases the spatial sampling positions in the network by introducing additional offsets, allowing the receptive field to adaptively adjust according to the scale and shape of the target, and achieving multi-scale dense feature extraction. The deformable convolution is presented in Equation (1) [16]:


$$\begin{array}{c} y\left(p_{n}\right)=\sum_{p_{n}\subset R}w\left(p_{n}\right)\cdot x(p+p_{n}+\Delta p_{n}s\left(\left(p\right)\right). \end{array}$$ (1)

**Fig 3:**
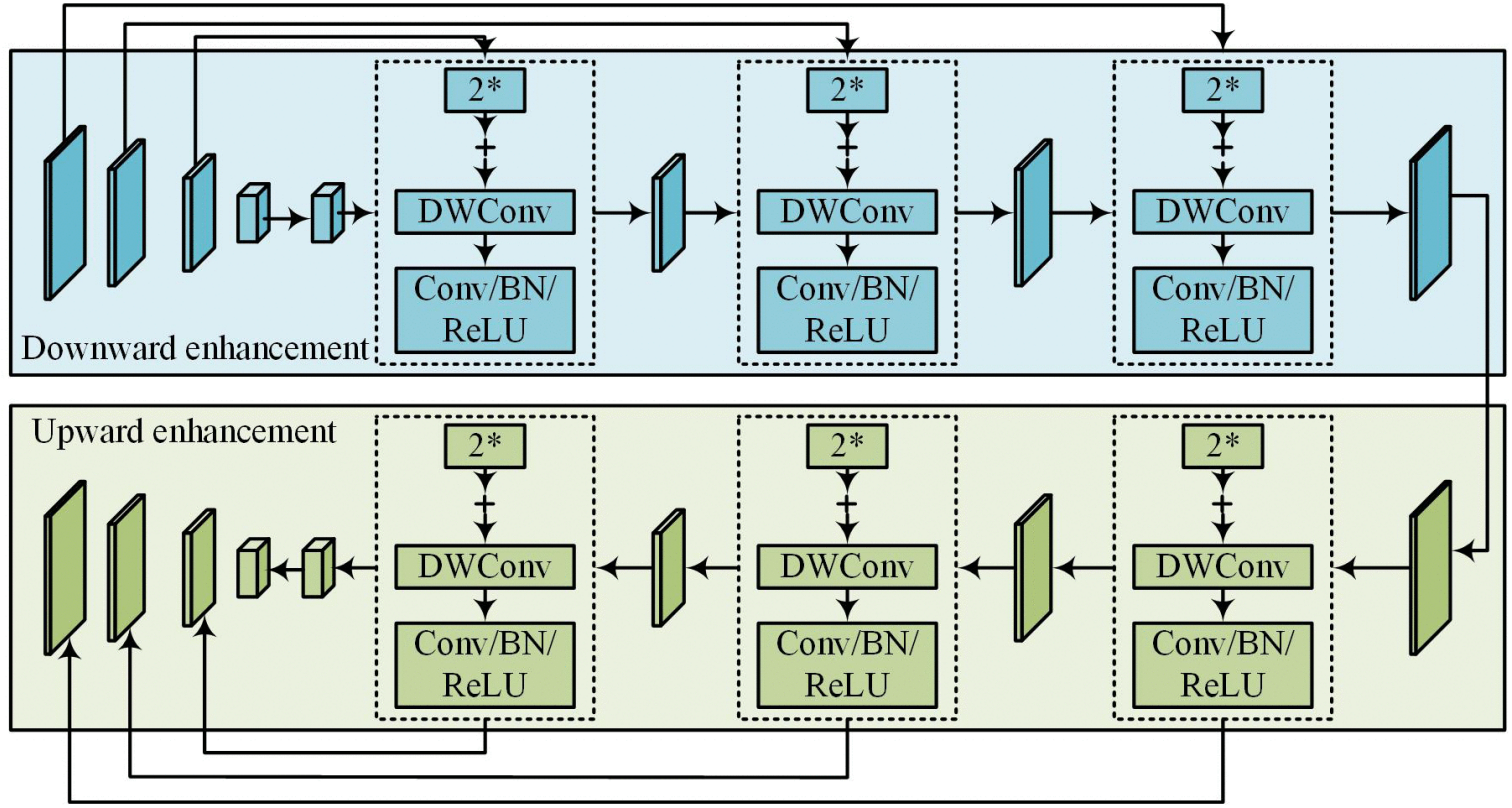
Structure of FPEM.

In Equation (1), y(p) signifies the value of the output feature map at position $p_{n}$. $w\left(p_{n}\right)$ signifies the weight of the convolution kernel at position $p_{n}$. x(.) signifies the input feature map. $\Delta p_{n}$ signifies the offset. s(p) represents the scaling factor. Although deformable convolution can effectively extract multi-scale features, DBNet still has weak feature expression ability. Therefore, FPEM is introduced to enhance feature expression ability. The structure of FPEM is shown in Fig 3.

In Fig 3, FPEM consists of two stages: upward enhancement and downward enhancement. In the upward enhancement stage, the input feature map is up-sampled twice, added to the previous feature map, and then subjected to 3*3 depth separable convolution and 1*1 ordinary convolution. The downward enhancement stage is similar to the upward enhancement stage, except that its depth separable convolution step size is 2. After the above operation, multiple feature maps of different scales can be obtained. After up-sampling and concatenation, multi-scale feature maps can generate probability maps and threshold maps. Based on the differentiable function that approximates a step function, the probability graph is transformed into an approximate binary graph. The differentiable binarization is presented in Equation (2) [17]:


$$\begin{array}{c} {\hat{B}}_{i,j}=\frac{\text{1}}{1+e^{-k(P_{i,j}-T_{i,j})}}. \end{array}$$ (2)

In Equation (2), ${\hat{B}}_{i,j}$ represents a binary graph. k represents the gain factor, with a value of 50. $P_{i,j}$ represents the probability graph. $T_{i,j}$ represents the threshold map. Table 1 displays the key parameters of DBNet-FPEM.

**Table 1:** Key parameters of DBNet-FPEM.

Name	Convolutional kernel size/stride	Number of convolution kernels
Conv 1	7*7/2	64
Conv 2	3*3/1,3*3/1	64
Conv 3	(3*3/2,1*1/2,3*3/1),(3*3/1,3*3/1)	128
Conv 4	(3*3/2,1*1/2,3*3/1),(3*3/1,3*3/1)	256
Conv 5	(3*3/2,1*1/2,3*3/1),(3*3/1,3*3/1)	512
Upward enhancement 1	3*3/1,1*1/1	128
Upward enhancement 2	3*3/2,1*1/1	128
Downward enhancement 1	3*3/1,1*1/1	128
Downward enhancement 2	3*3/2,1*1/1	128

According to Table 1, the convolution kernel sizes of each module include two types: 3*3 and 1*1, with a stride of 1 or 2. For the loss function of the model, it consists of probability graph loss, binary graph loss, and threshold graph loss. The loss function of the probability graph is shown in Equation (3):


$$\begin{array}{c} L_{P}=\sum_{i\in S_{l}}l_{i}\;logp_{i}+(1-l_{i})log(1-p_{i}). \end{array}$$ (3)

In Equation (3), $L_{P}$ represents the loss function of the probability graph. $S_{i}$ represents the resampled dataset. $l_{i}$ represents the true label. $p_{i}$ represents the predicted value of pixel probability. The loss function of the binary graph is shown in Equation (4):


$$\begin{array}{c} L_{B}=\sum_{i\in B_{d}}1-\frac{2\left|\overset{P}{\underset{i}{b}}\cap \overset{R}{\underset{i}{b}}\right|}{\left|\overset{P}{\underset{i}{b}}\right|+\left|\overset{R}{\underset{i}{b}}\right|}. \end{array}$$ (4)

In Equation (4), $L_{B}$ represents the loss function of the binary graph. $B_{d}$ represents the set of pixels in the binary image. $\overset{P}{\underset{i}{b}}$ signifies the predicted value of the binary graph. $\overset{R}{\underset{i}{b}}$ signifies the true value of the binary graph. The loss function of the threshold graph is shown in Equation (5):


$$\begin{array}{c} L_{T}=\sum_{i\in R_{d}}\left|l_{T,i}-p_{T,i}\right|. \end{array}$$ (5)

In Equation (5), $L_{T}$ represents the loss function of the threshold map. $R_{d}$ represents the set of pixels within the expanded boundary of the threshold map. $l_{T,i}$ signifies the true value of the threshold icon label. $p_{T,i}$ signifies the predicted value of the threshold map. The loss function of DBNet-FPEM is presented in Equation (6):


$$\begin{array}{c} L=L_{P}+\alpha \times L_{B}+\beta \times L_{T}. \end{array}$$ (6)

In Equation (6), L signifies the overall loss function. α and β both represent equilibrium factors, with values of 5 and 10, respectively. A handwritten tax receipt detection method is constructed through the above method, which can accurately locate the text.

### 2.2. Handwritten tax receipt recognition method based on improved CRNN

**Fig 4:**
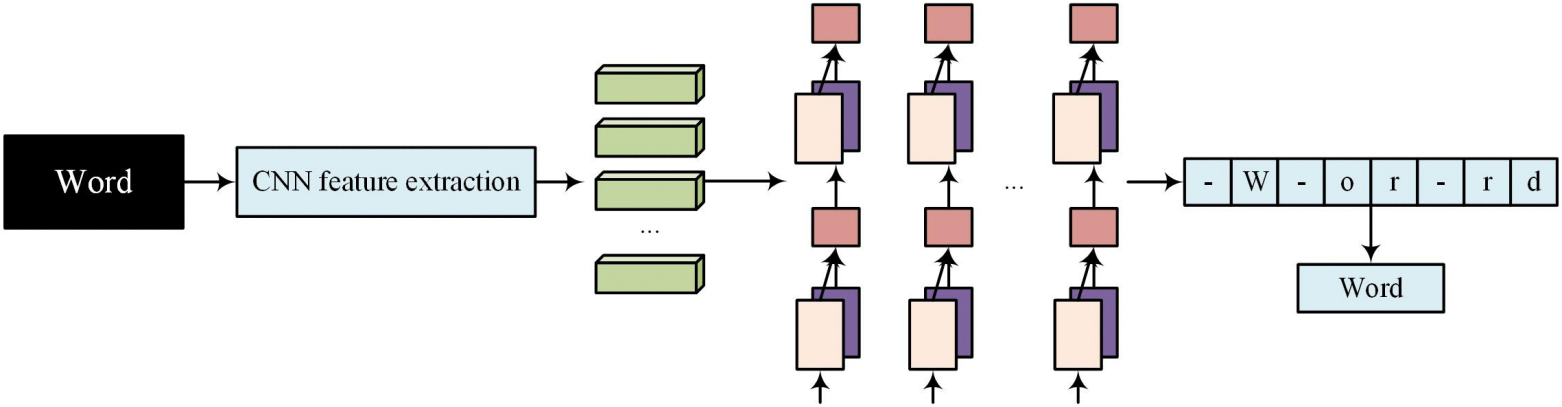
Handwritten tax receipt recognition model based on improved CRNN.

Although the above method can accurately locate the text area of handwritten tax receipts, the recognition difficulty is often high due to the large differences in handwriting and text length of handwritten receipts. Therefore, to accurately recognize handwritten tax receipts, a recognition model based on CRNN is proposed. Compared with other algorithms, CRNN can handle sequences of any length without the need for pre-defined dictionaries, and it also has high computational efficiency and strong robustness [18]. Although CRNN can handle longer text sequences, it has strong sensitivity to skewed text and limited recognition ability for artistic and handwritten fonts [19,20]. Therefore, to improve the handwritten font recognition accuracy of CRNN, DenseNet is used to improve it. The handwritten tax receipt recognition model based on improved CRNN is presented in Fig 4.

**Fig 5:**
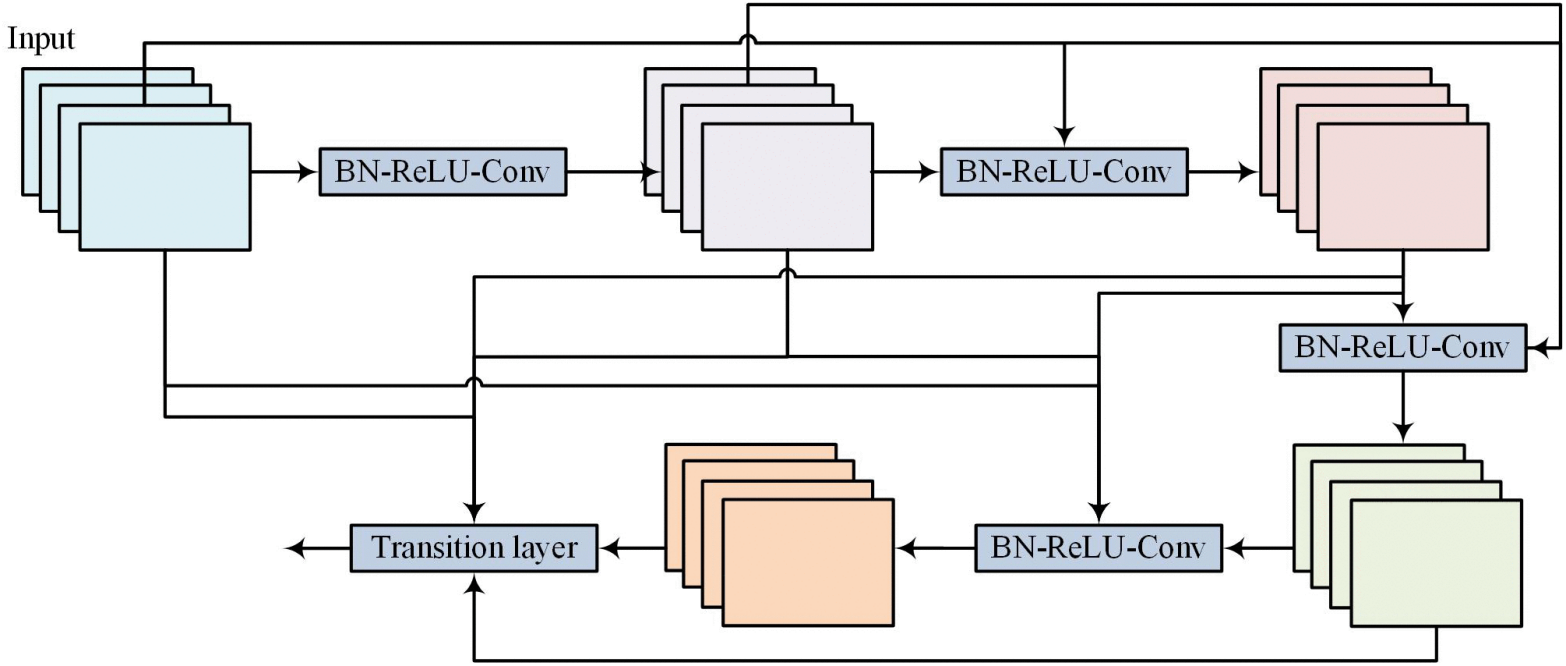
Structure of DenseNet.

In Fig 4, the improved CRNN has a feature sequence layer, a loop layer, and a transcription layer. The feature sequence layer extracts features from the input image. The loop layer is responsible for predicting and learning feature sequences, and outputting the predicted labels (true values). The transcription layer converts the obtained series of label distributions into the final label sequence. In the feature sequence layer, the original CRNN model extracts image features through CNN. However, due to the diverse features of handwritten fonts, the expressive ability of CNN is weak. Therefore, the DenseNet module replaces Conv3 to Conv9 in the original CNN to reconstruct a high-performance feature extraction network. The DenseNet structure is shown in Fig 5.

**Fig 6:**
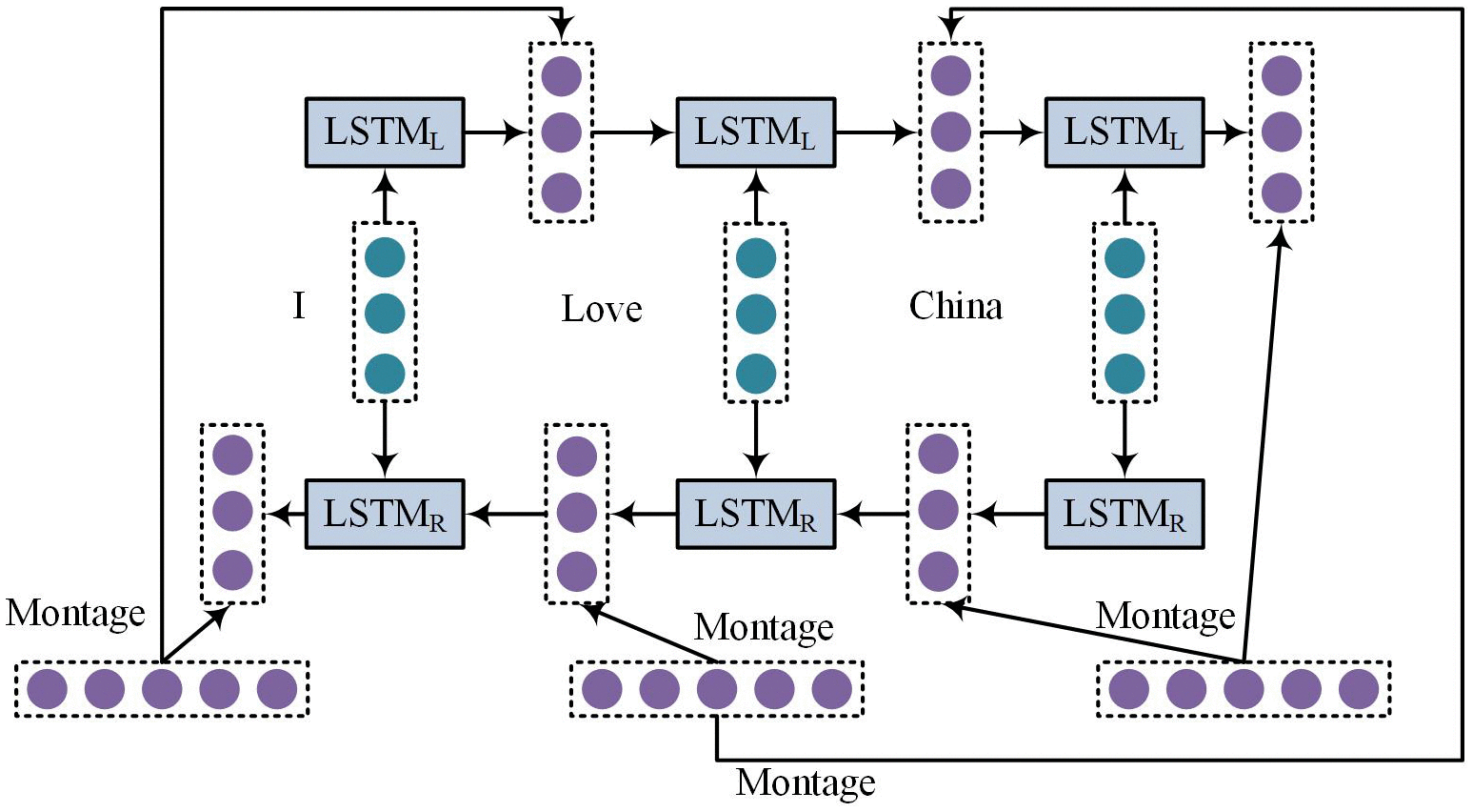
Structure of BiLSTM.

In Fig 5, DenseNet involves dense blocks and transition layers. The dense block contains multiple convolutional layers, each of which is densely connected to all previous layers and uses a smaller growth rate to control the number of new features generated by each layer. The transition layer is located between adjacent dense blocks and is used for down-sampling, which involves batch normalization, 1*1 convolution, and average pooling. When collecting image information, the width information is easily lost due to the small aspect ratio of handwritten text images. Therefore, to preserve the width information of the image, the study replaces the original pooling layer with a 1*2 average pooling layer. The reason for choosing 1*2 average pooling is that the aspect ratio of handwritten tax receipt text images is small, and the horizontal width information is crucial for character differentiation. The 1*2 kernel size only down-samples vertically, which can preserve the horizontal texture and character boundary information to the greatest extent possible, avoiding the loss of width features. If maximum pooling is used, it is easy to lose continuous stroke features due to local pixel extremum of handwritten strokes, especially for characters with stroke adhesion and sloppy writing, which will exacerbate feature fragmentation. Average pooling can smoothly extract regional features, and improve robustness to stroke deformation and blurring, which is more suitable for character recognition scenarios of handwritten tax receipts. In the recurrent layer, the RNN predicts characters based on feature sequences. However, the text has a strong correlation before and after, and there is a long-term dependency problem. Therefore, to accurately predict text, the RNN used is a BiLSTM network. BiLSTM can simultaneously consider past and future information in a sequence, capturing bidirectional dependencies. Meanwhile, BiLSTM can also combine forward and backward information to better capture long-term dependencies. The structure of BiLSTM is presented in Fig 6.

In Fig 6, BiLSTM consists of a forward LSTM layer and a backward LSTM layer. The forward LSTM layer is responsible for forward processing of the sequence, while the backward LSTM layer is responsible for backward processing of the sequence. At each time step, both LSTM layers will generate a hidden state, which will be merged through concatenation to form a single representation containing bidirectional information. This merged representation can provide richer features for each time point in the sequence, as it simultaneously considers the context of the sequence. The forget gate of BiLSTM is shown in Equation (7) [21]:


$$\begin{array}{c} f_{t}=\sigma (W_{f}\cdot [h_{t-1},x_{t}]+b_{f}). \end{array}$$ (7)

In Equation (7), $f_{t}$ signifies the output of the forget gate. $W_{f}$ signifies the weight of the forget gate. $b_{f}$ signifies the bias of the forget gate. σ signifies the Sigmoid activation function. $h_{t-1}$ signifies the hidden state of the previous time step. $x_{t}$ signifies the input of the current moment. It is necessary to select activation functions for module function adaptation in the model: BiLSTM gating uses Sigmoid to achieve 0–1 information filtering and adapt to temporal features. DenseNet and convolutional layers use ReLU to alleviate gradient vanishing and improve feature extraction efficiency. If replaced with functions such as Tanh and Swish, gating can easily lose key information in text sequences or increase computational complexity, which contradicts the high-precision and high-speed requirements of handwritten note recognition. Therefore, the activation function is crucial for model performance. The input gate is shown in Equation (8) [22]:


$$\begin{array}{c} \left\{ \begin{array}{c} @l@i_{t}=\sigma (W_{i}\cdot [h_{t-1},x_{t}]+b_{i})\\ {\tilde{C}}_{t}=tanh(W_{C}\cdot [h_{t-1},x_{t}]+b_{C}) \end{array}\right.. \end{array}$$ (8)

In Equation (8), $i_{t}$ represents the output of the input gate. $W_{i}$ represents the weight of the forget gate. $b_{i}$ represents the bias of the forget gate. ${\tilde{C}}_{t}$ represents the candidate cell state. $W_{C}$ and $b_{C}$ represent the weights and biases of candidate cell states, respectively. The cell state is shown in Equation (9) [23]:


$$\begin{array}{c} C_{t}=f_{t}*C_{t-1}+i_{t}*{\tilde{C}}_{t}. \end{array}$$ (9)

In Equation (9), $C_{t}$ signifies the cell state at the current moment. The output gate is presented in Equation (10) [24]:


$$\begin{array}{c} \left\{ \begin{array}{c} @l@o_{t}=\sigma (W_{o}\cdot [h_{t-1},x_{t}]+b_{o})\\ h_{t}=o_{t}*tanh\left(C_{t}\right) \end{array}\right.. \end{array}$$ (10)

In Equation (10), $o_{t}$ signifies the output of the output gate. $W_{o}$ signifies the weight of the output gate. $b_{o}$ signifies the bias of the output gate. In the transcription layer, considering the variable length of text sequences, the algorithm used is the Connectionist Temporal Classification (CTC) algorithm, whose core idea is to solve the matching problem between input and output sequences. This algorithm proposes an alignment method that divides the output sequence into multiple time periods and achieves sequence alignment by establishing certain processing rules. The input-output sequence of CTC algorithm is shown in Equation (11):


$$\begin{array}{c} \left\{ \begin{array}{c} @l@I=\left(i^{\text{1}},i^{\text{2}},\cdots ,i^{T}\right)\\ O=\left(o^{\text{1}},o^{\text{2}},\cdots ,o^{T}\right) \end{array}\right.. \end{array}$$ (11)

In Equation (11), I and $i^{T}$ represent the input sequence and its constituent elements, respectively. O and $o^{T}$ respectively represent the output sequence and its constituent elements. For a given input sequence, the posterior probability of its output is shown in Equation (12):


$$\begin{array}{c} p\left(o\right|i)=\sum_{F\left(r\right)=o}P(r\left|i\right). \end{array}$$ (12)

In Equation (12), p(o|i) represents the posterior probability of the output to the input. r represents the path corresponding to the intermediate result. The posterior probability of the output path is shown in Equation (13):


$$\begin{array}{c} p\left(r|i\right)=\prod_{i=1}^{T}\overset{t}{\underset{r_{t}}{z}}. \end{array}$$ (13)

In Equation (13), p(r|i) signifies the posterior probability of the output path to the input. $\overset{t}{\underset{r_{t}}{z}}$ represents the probability of selecting character $r_{t}$ at the current moment. At this point, the posterior probability of the output to the input can be rewritten as Equation (14):


$$\begin{array}{c} p\left(o|i\right)=\sum_{F\left(r\right)=o}\prod_{t=1}^{T}\overset{t}{\underset{r_{t}}{z}}. \end{array}$$ (14)

The above CTC algorithm can achieve alignment between input and output sequences to improve the recognition speed. The loss function of the model is shown in Equation (15):


$$\begin{array}{c} L_{CRNN}=\sum_{\left(F,L\right)\in D}-log\text{\textbackslash{}hspace\{0.17em}p\left(L|F\right)\} \end{array}$$ (15)

In Equation (15), $L_{CRNN}$ represents the loss function of the model. F represents the feature sequence. L signifies the true label of the training set images. D signifies the training set. p(L|F) signifies the conditional probability.

## 3. Results

The research comparison algorithms all select mainstream advanced models in the current scene text detection and recognition field. The detection class selects the Text Detection model based on Segformer and Enhanced Feature Pyramid (TDMSEFP), Improved Fourier Contour Embedding Network (IFCENet), Identification class selection Dual-Stream Network Fusing Spatial and Frequency Domain Features (DSNFSDF), and Multi-modal Network based on Visual Attention and Semantic Perception (MMNVASC). All benchmark methods proposed by top journals/conferences in the same field ensure fairness and effectiveness in comparison. All comparison algorithms and the proposed model are optimized in the same software and hardware environment, using grid search method to optimize hyperparameters such as learning rate and batch size, and determining the final parameters based on the optimal performance of the validation set. The experiment uses the Total Text dataset, which includes 1,555 images covering horizontal, multi-directional, and curved text, 11,165 annotated text instances, and is adapted to the complex text features of handwritten tax receipts.

### 3.1. Test results of handwritten tax receipt detection model

**Fig 7:**
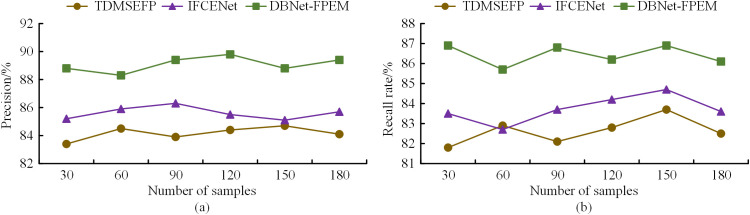
Precision and recall rate of various models (a) Precision (b) Recall rate.

To validate the handwritten tax receipt detection model, it is tested and compared with the TDMSEFP and the IFCENet. The dataset used in the experiment is the Total-Text dataset, which consists of 1,555 images covering over three different text directions (horizontal, multi-directional, and curved). The CPU is Intel Xeon E 2224, the operating system is Windows 10, and the deep learning framework is PaddlePaddle 2.4. In the experiment, the learning rate and batch size of DBNet-FPEM are 0.001 and 16, respectively. The epoch is 1,200, and the optimizer and regularization methods are Adam and L2, respectively. The binarization threshold and vatti expansion coefficient are 0.7 and 1.5, respectively. The detection precision and recall rate of different models are shown in Fig 7.

**Fig 8:**
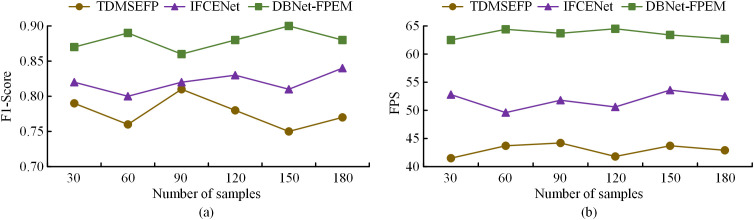
F1-Score and FPS of different models (a) F1-Score (b) FPS.

In Fig 7(a), the precision of TDMSEFP and IFCENet was the highest at 84.7% and 86.3%, and the lowest at 83.4% and 85.1%, respectively. The average detection precision was 84.2% and 85.6%, respectively. The detection precision of DBNet-FPEM was the lowest at 88.3%, with a precision up to 89.1%, far higher than that of other models. In Fig 7(b), DBNet-FPEM had a higher recall rate, with the lowest recall rate of 85.7% and an average recall rate of 86.4%. The highest recall rates for TDMSEFP and IFCENet were 83.7% and 84.7%, respectively, with average recall rates of 82.6% and 83.7%, respectively. DBNet-FPEM has higher text detection precision. The F1-Score and Frames per Second (FPS) of different models are shown in Fig 8.

**Fig 9:**
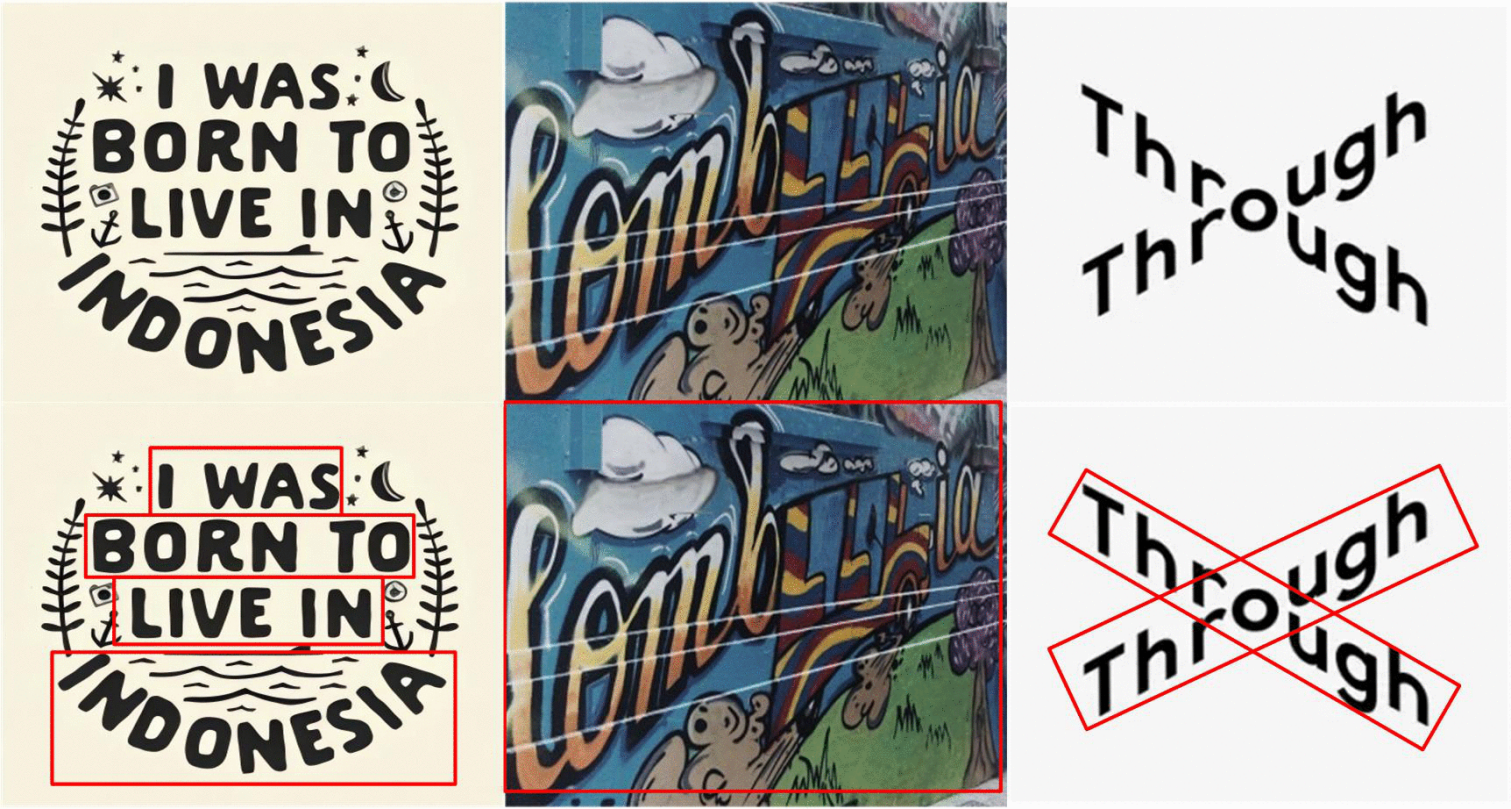
Text detection results of DBNet-FPEM.

From Fig 8(a), the F1-Score of TDMSEFP and IFCENet was the highest, at 0.81 and 0.84, and the lowest at 0.75 and 0.80, respectively. The average F1-Score was 0.78 and 0.82, respectively. The lowest F1-Score of DBNet-FPEM was 0.86, and the average F1-Score was 0.88, far higher than that of other models. From Fig 8(b), the FPS of TDMSEFP and IFCENet was the highest at 44.2 and 53.6, and the lowest at 41.5 and 49.6, respectively. The average FPS was 43.0 and 51.8, respectively. The lowest FPS of DBNet-FPEM was 62.5, and the average FPS was 63.5, which was also much higher than that of other models. The above results indicate that the overall performance of DBNet-FPEM is superior to other models. The text detection results of DBNet-FPEM are presented in Fig 9.

In Fig 9, DBNet-FPEM could accurately detect text in different situations, even if the text was tilted or deformed. This proves that DBNet-FPEM has strong deformation resistance and can accurately locate text in different situations. To verify the impact of FPEM on model performance, ablation experiments are conducted, as presented in Table 2.

**Table 2:** Results of ablation experiment.

Quantity of FPEM	Precision/%	Recall/%	F1-Score	FPS
0	84.5	83.1	0.81	69.9
1	86.8	84.4	0.85	65.7
2	89.1	86.4	0.88	63.5
3	89.6	87.3	0.89	58.4
4	88.9	86.1	0.86	56.2
5	88.3	85.9	0.85	54.9

According to Table 2, as the number of FPEMs increased, their detection precision, recall, and F1-Score all first increased and then decreased, while FPS gradually decreased. When the number of FPEMs was 3, the detection precision, recall, and F1-Score were the highest, at 89.6%, 87.3%, and 0.89, respectively, but its FPS was only 58.4. When the number of FEPMs was 2, although their detection precision, recall, and F1-Score slightly decreased, the FPS was 63.5, much higher than that of three FPEMs. Therefore, when the FPEM is 3, its overall model performance is optimal.

### 3.2. Test results of handwritten tax receipt recognition model

**Fig 10:**
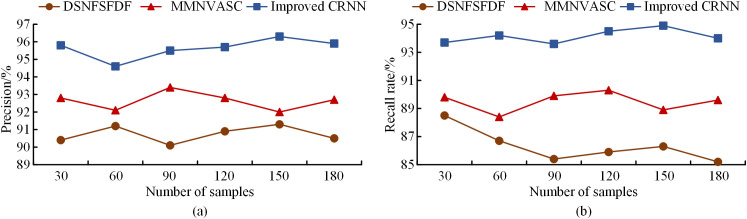
Recognition precision and recall rate of different models (a) Precision (b) Recall rate.

To verify the performance of the handwritten tax receipt recognition model proposed in the study, it is tested and compared with the DSNFSDF and the MMNVASC models. In the experiment, the epoch and batch sizes of the improved CRNN were 800 and 32, respectively, with learning rates of [0.0001, 0.001]. The software, hardware, and dataset used in the experiment are consistent with the previous text and will not be further elaborated. The recognition precision and recall rate of different models are shown in Fig 10.

**Fig 11:**
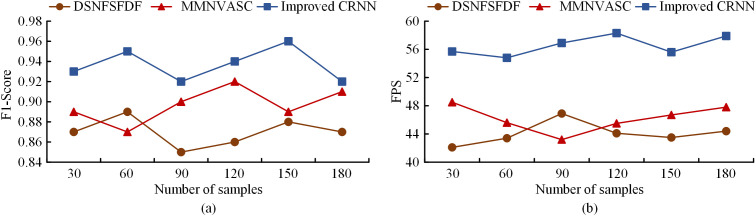
F1-Score and FPS of different models (a) F1-Score (b) FPS.

According to Fig 10(a), the recognition precision of DSNFSDF and MMNVASC was the highest at 91.3% and 93.4%, and the lowest at 90.1% and 92.0%, respectively. The average recognition precision was 90.7% and 92.6%, respectively. The lowest recognition precision of improved CRNN was 94.6%, and the average recognition precision was as high as 95.6%, far higher than that of other models. According to Fig 10(b), the improved CRNN had a higher recall rate, with the lowest recall rate of 93.6% and an average recall rate of 94.2%. The highest recall for DSNFSDF and MMNVASC was not more than 91%, with average recall rates of 86.3% and 89.5%, respectively. The improved CRNN has higher text recognition precision. The F1-Score and FPS of different models are shown in Fig 11.

**Fig 12:**
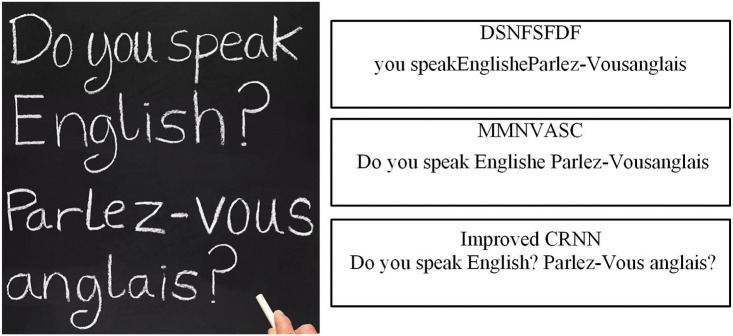
Identification results of different models.

From Fig 11(a), the F1-Score of DSNFSDF and MMNVASC was the highest at 0.89 and 0.92, and the lowest at 0.85 and 0.87, respectively. The average F1-Score was 0.87 and 0.90, respectively. The lowest F1-Score of the improved CRNN was 0.92, and the average F1-Score was 0.94, far higher than that of other models. From Fig 11(b), the FPS of DSNFSDF and MMNVASC was the highest at 46.9 and 48.5, the lowest at 42.1 and 43.2, and the average FPS was 44.1 and 46.2, respectively. The improved CRNN had a minimum FPS of 54.8 and an average FPS of 56.5, which was also much higher than that of other models. The improved CRNN has better recognition speed and accuracy than other models. The recognition results of different models are shown in Fig 12.

From Fig 12, there were a large number of misspellings in the recognition results of DSNFSDF, which led to significant changes in the text meaning. Although the recognition results of MMNVASC showed obvious reduction in the number of characters and the text meaning was basically clear, it still had a significant impact on the reading experience. There was no typo error in the recognition results of improved CRNN. The improved CRNN had better text recognition performance. To verify the performance of the improved CRNN, ablation experiments are conducted. The experimental results are shown in Table 3.

**Table 3:** Results of ablation experiment.

DenseNet	1*2 average pooling	BiLSTM	Precision/%	F1-Score
×	×	0	81.6	0.79
×	√	0	83.5	0.81
×	×	1	82.8	0.79
×	√	1	86.7	0.83
√	×	0	84.8	0.81
√	×	1	87.2	0.86
√	×	2	89.6	0.88
√	√	0	85.7	0.82
√	√	1	92.4	0.89
×	×	2	89.6	0.87
×	√	2	93.3	0.91
√	√	2	95.6	0.94

According to Table 3, the recognition precision significantly improved after introducing DenseNet and 1*2 average pooling. In addition, as the BiLSTM increased, the recognition precision also gradually improved. When DenseNet, 1*2 average pooling, and BiLSTM were not present, the recognition precision was only 81.6%. When DenseNet and 1*2 average pooling were introduced, and the number of BiLSTM modules was 2, the recognition precision increased to 95.6%. Clearly, DenseNet, 1*2 average pooling, and BiLSTM have a synergistic effect on the model recognition performance. The improvement of a single module has a limited increase in precision, only 2% ~ 8%. When the three were combined, the performance was optimal with a precision of 95.6%. The increase in the number of BiLSTM modules has the most significant impact on performance gain, with 2-layer BiLSTM improving precision by about 10% compared to 0-layer, confirming the key role of bidirectional temporal feature capture in handwriting recognition. The recognition precision of improved CRNN was significantly affected by DenseNet, 1*2 average pooling, and BiLSTM. To further validate the generalization ability of the proposed algorithm, it was compared with cutting-edge technologies on the Total Text and SCUT-IARAC (a dedicated dataset for handwritten receipts, including 2,000 images of tax/financial handwritten receipts) datasets. The core evaluation indicators of each model are shown in Table 4.

**Table 4:** Generalization ability test results.

Model Type	Model	Dataset	Precision/%	F1-Score
Text detection	TDMSEFP	Total-Text	84.2	0.78
		SCUT-IRAC	83.8	0.76
	IFCENet	Total-Text	85.6	0.82
		SCUT-IRAC	84.2	0.81
	DBNet-FPEM	Total-Text	89.1	0.88
		SCUT-IRAC	90.5	0.89
Text recognition	DSNFSDF	Total-Text	90.7	0.87
		SCUT-IRAC	89.1	0.84
	MMNVASC	Total-Text	92.6	0.90
		SCUT-IRAC	90.9	0.88
	Improved CRNN	Total-Text	95.6	0.94
		SCUT-IRAC	96.8	0.96

According to Table 4, in terms of detection performance, on the Total Text dataset, the precision (89.1%) and F1-Score (0.88) of the proposed DBNet-FPEM were significantly higher than those of TDMSEFP (84.2%, 0.78) and IFCENet (85.6%, 0.82). On the handwritten bill dedicated dataset SCUT-IARAC, its performance has been further improved to Precision 90.5% and F1-Score 0.89, with more obvious advantages. Its precision was 90.5% and the F1-Score was 0.89, making the advantage even more apparent. In terms of recognition performance, on the Total Text dataset, the precision (95.6%) and F1-Score (0.94) of Improved CRNN far exceeded those of DSNFSDF (90.7%, 0.87) and MMNVASC (92.6%, 0.90). On the SCUT-IARAC dataset, the precision was 96.8% and F1-Score was 0.96. Overall, the proposed algorithm outperforms the comparison model in both general scenarios and handwritten tax receipt specific scenarios, and its performance gains are more prominent on specialized datasets, demonstrating its generalization ability and scene adaptability.

## 4. Discussion

In the field of tax management, efficient and accurate input of handwritten receipts is a key link in the digitization and modernization of tax declaration management. The traditional manual input method is not only inefficient, but also prone to errors, greatly limiting the efficiency and accuracy of tax management. An automatic handwritten tax receipt recognition method is developed that can significantly reduce workload, improve the efficiency and accuracy of tax returns, and promote the digital transformation of tax management [25]. However, due to the significant differences in handwritten fonts, there may be strokes and interference between characters, and the complex and diverse layout of receipts makes it difficult to recognize handwritten tax receipts [26]. Therefore, a handwritten tax receipt recognition method based on DBNet and CRNN is proposed. This method first combines DBNet with FPEM to detect the text of handwritten receipts to precisely locate the text. The improved CRNN is used to recognize the text of handwritten receipts.

In terms of text detection, DBNet-FPEM achieved detection precision, recall, and F1-Score of 89.1%, 86.4%, and 0.88, respectively, significantly higher than that of other comparison models. Regarding the text detection, Liao et al. built a detection method based on adaptive scale fusion and DBNet, which improved scale robustness by adaptively fusing features with various scales [27]. Compared with the above methods, DBNet-FPEM had better detection precision and speed. This is because FPEM enhances the model’s adaptability to texts with different sizes through multi-scale feature fusion, while deformable convolution further improves the model’s detection accuracy for complex text layouts by adaptively adjusting the shape and scale of the convolution kernel [28]. In addition, the ablation experiment results showed that when the FPEM was 2, the model achieved a balance in detection precision, recall, and F1-Score, which further verified the key role of FPEM in improving model performance.

In terms of text recognition, the improved CRNN significantly enhanced feature extraction capability by introducing DenseNet module and 1 × 2 average pooling layer, and combined with BiLSTM to optimize the ability to capture long-term dependencies of text sequences. The improved CRNN outperformed other advanced models in recognition precision, recall rate, and F1-Score, with an average recognition precision of 95.6%, an average recall rate of 94.2%, and an average F1-Score of 0.94. The improved CRNN model had higher precision and robustness in handling complex handwritten tax receipts. Coquenet et al. built a unified end-to-end model based on mixed attention for handwritten text recognition. This model utilized an encoder to generate feature maps from text images. Then, a vertically weighted mask was repeatedly generated through the attention module to focus on the current text line features [29]. The improved CRNN had higher recognition precision and speed. This is because the DenseNet module significantly enhances the feature extraction ability, especially when dealing with complex features of handwritten fonts. In addition, the 1 × 2 average pooling layer effectively preserves the width information of the image, further optimizing the recognition precision. The BiLSTM optimizes the ability to capture long-term dependencies on text sequences, enabling the model to predict text content more accurately [30].

The proposed algorithm has strong scalability and can be extended from both model architecture and practical applications. This is because the algorithm adopts a modular design, where the DBNet-FPEM detection module and the improved CRNN recognition module are independent of each other and can be replaced, upgraded, or integrated with other OCR algorithm modules to meet the customized needs of different recognition scenarios. The core multi-scale feature fusion and bidirectional temporal feature capture mechanism are not limited to tax bills, and can be transferred to various handwritten bill recognition tasks such as invoices, certificates of deposit, and customs declarations by fine-tuning the annotated dataset with small samples. In addition, the model supports the expansion of computing power adaptability, which can be achieved through adjusting the number of convolutional layers and pooling methods to achieve lightweight deployment on hardware platforms with different computing power.

In summary, the handwritten tax receipt recognition method based on improved DBNet and CRNN performs well in both detection and recognition performance, and has important practical application value. In addition, this method significantly improves the efficiency and accuracy of tax management, promotes the development and innovation of OCR technology, and has important practical significance and far-reaching impact.

## 5. Conclusion and future direction

To recognize handwritten tax receipts and enhance tax management efficiency, this paper proposes a handwritten tax receipt recognition method based on improved DBNet and CRNN. The method integrates FPEM and deformable convolution to optimize DBNet for precise text detection. DenseNet, 1 × 2 average pooling, and dual-BiLSTM are combined to strengthen the feature extraction and sequence modeling capabilities of CRNN. Experimental results show that the improved DBNet achieves 89.1% average detection precision, 86.4% recall, and 0.88 F1-Score, while the optimized CRNN obtains 95.6% average recognition precision, 94.2% recall, and 0.94 F1-Score, both outperforming mainstream models and exhibiting strong robustness in complex handwriting scenarios. This work provides an effective technical solution for the digital processing of handwritten tax receipts, reduces manual workload, and promotes the intelligent transformation of tax administration. This method still has limitations: it relies on high-quality annotated data, resulting in decreased accuracy in recognizing handwritten characters that are severely blurry, soiled, or stuck. Multi-module fusion increases model complexity and makes real-time deployment of low computing edge devices difficult, only applicable to handwritten tax receipts, and not verified on other types of tax documents. Future research will focus on model lightweight design, enhancing the anti-interference ability of fuzzy and connected handwriting, and constructing a multi-scenario tax receipt dataset to further improve generalization ability and practical deployment performance.
